# Clinical Validation of Respiratory Rate Estimation Using Acoustic Signals from a Wearable Device

**DOI:** 10.3390/jcm13237199

**Published:** 2024-11-27

**Authors:** Rawan S. Abdulsadig, Nikesh Devani, Sukhpreet Singh, Zaibaa Patel, Renard Xaviero Adhi Pramono, Swapna Mandal, Esther Rodriguez-Villegas

**Affiliations:** 1 Wearable Technologies Lab, Department of Electrical and Electronic Engineering, Imperial College London, London SW7 2BT, UK; 2 Thoracic Medicine, Royal Free London NHS Foundation Trust, London NW3 2QG, UK

**Keywords:** respiratory rate, digital health, wearable technology, validation study, AcuPebble

## Abstract

**Objectives**: Respiratory rate (RR) is a clinical measure of breathing frequency, a vital metric for clinical assessment. However, the recording and documentation of RR are considered to be extremely poor due to the limitations of the current approaches to measuring RR, including capnography and manual counting. We conducted a validation of the automatic RR measurement capability of AcuPebble RE100 (Acurable, London, UK) against a gold-standard capnography system and a type-III cardiorespiratory polygraphy system in two independent prospective and retrospective studies. **Methods**: The experiment for the prospective study was conducted at Imperial College London. Data from AcuPebble RE100 (Acurable, London, UK) and the reference capnography system (Capnostream™35, Medtronic, Minneapolis, MN, USA) were collected simultaneously from healthy volunteers. The data from a previously published study were used in the retrospective study, where the patients were recruited consecutively from a standard Obstructive Sleep Apnea (OSA) diagnostic pathway in a UK hospital. Overnight data during sleep were collected using the AcuPebble SA100 (Acurable, London, UK) sensor and a type-III cardiorespiratory polygraphy system (Embletta MPR Sleep System, Natus Medical, Pleasanton, CA, USA) at the patients’ homes. Data from 15 healthy volunteers were used in the prospective study. For the retrospective study, 150 consecutive patients had been referred for OSA diagnosis and successfully completed the study. **Results**: The RR output of AcuPebble RE100 (Acurable, London, UK) was compared against the reference device in terms of the Root Mean Squared Deviation (RMSD), mean error, and standard deviation (SD) of the difference between the paired measurements. In both the prospective and retrospective studies, the AcuPebble RE100 algorithms provided accurate RR measurements, well within the clinically relevant margin of error, typically used by FDA-approved respiratory rate monitoring devices, with the RMSD under three breaths per minute (BPM) and mean errors of 1.83 BPM and 1.4 BPM, respectively. **Conclusions**: The evaluation results provide evidence that AcuPebble RE100 (Acurable, London, UK) algorithms produce reliable results and are hence suitable for overnight monitoring of RR.

## 1. Introduction

Respiratory rate (RR) is a clinical measure of breathing frequency, often used to assess patient distress and respiratory degradation [1,2,3]. RR can indicate a patient’s ability to maintain homeostatic control [4], thus acting as a predictor of cardiopulmonary arrest, patient deterioration, or even death [1,5,6]. Similar to heart rate (HR), peripheral blood oxygen saturation (SpO_2_), and temperature, RR is a vital metric for clinical assessment and is part of the National Early Warning Score (NEWS2) that needs to be routinely recorded [4]. However, out of these metrics, the recording and documentation of RR is considered to be the most challenging. This is a well-recognized issue [7,8] and could be attributed to the limitations of the current approaches to measuring RR, including capnography and manual counting [9]. Capnography, a gold standard for continuous RR monitoring, is administered using a nasal cannula measuring end-tidal CO_2_. However, capnography is primarily used for intubated patients [10]. In the absence of capnography, manual counting is performed by a clinician counting chest movements in 15–60 s windows to calculate the RR [11]. However, this is a time-consuming process, where the intended recording frequency is often not achieved and can be subject to counting error [12].

Despite the prevalence of continuous non-invasive HR or SpO_2_ monitors using pulse oximetry, only recently have continuous recording RR monitors become prominent. Sensing techniques such as photoplethysmography, acoustics, bio-impedance, inertial sensors, and even cameras have provided more accessible and user-friendly alternatives to continuous RR monitoring [13]. Remote RR monitoring shows promise in increasing access to out-of-clinic care, providing benefits for those suffering from respiratory illnesses such as asthma, pneumonia, Chronic Obstructive Pulmonary Disease (COPD), Obstructive Sleep Apnea (OSA), and, more recently, COVID-19 [14,15,16,17,18]. Other applications include identifying signs of infection post-surgery, home-dwelling elderly patients, and those at risk of heart failure [19].

Many wearable breathing rate monitors meet the clinical accuracy standards, obtaining CE and FDA approvals, as shown in Appendix A Table A2. However, despite possessing clinical-grade accuracy, many wearable systems suffer from low compliance, with a significant factor being poor usability, along with human factor considerations [20,21]. Referring to Appendix A Table A2, the MightySat Rx (Masimo, Irvine, CA, USA) device, which uses photoplethysmography (PPG), achieves ±3 BPM accuracy over a 4–70 BPM range yet is limited to spot measurements. In contrast, the Masimo Root Monitoring System and Accessories (Masimo, Irvine, CA, USA), which also employs PPG, is designed for continuous monitoring in both adult and pediatric populations within clinical environments but is restricted to stationary conditions. The Philips Wearable Biosensor-G5 (Philips Healthcare, Best, The Netherlands), which combines ECG and accelerometry sensors, provides continuous monitoring at ±3 BPM accuracy in the range of 3–40 BPM but is limited to clinical settings. Thoracic impedance, as used in SimpleSENSE (Nanowear, Brooklyn, NY, USA), provides high accuracy (±2 BPM within 6–22 BPM), but this device also faces limitations, requiring stationary conditions. On the other hand, Masimo’s Radical-7 Pulse Co-Oximeter and Accessories (Masimo, Irvine, CA, USA), covering various settings and populations, including neonates, supports both spot and continuous RR monitoring at ±3 BPM accuracy across 4–70 BPM.

To this end, the main objective of this study is to validate the RR automatic algorithms of AcuPebble RE100 (Acurable, London, UK) against gold-standard capnography and cardiorespiratory polygraphy systems. AcuPebble RE100 is a physiological research platform consisting of a sensing device that is extremely easy to use and deploy [22] (equivalent to the FDA-approved AcuPebble SA100 (Acurable, London, UK) [23]) and a software package that can provide as outputs both the known physiological parameters and specific signal processing features that can be used for new algorithm development. The sensing device is a tiny wearable electronic technology weighing 7 g, measuring 2.9 cm in diameter and 1.4 cm in height, with a usability study in [22] showing the ease of use of the device and its accompanying app. The AcuPebble system was conceived to be used by patients without the need for intensive face-to-face training by a healthcare professional. The objective of this work is to validate the RR output performance of AcuPebble RE100 (Acurable, London, UK) since RR is known to be one of the more complex physiological parameters to accurately obtain automatically, both under controlled and extreme real-world conditions.

## 2. Materials and Methods

### 2.1. Study Design and Setting

Data from two studies were used to evaluate the performance of AcuPebble RE100 (Acurable, London, UK) RR output:

- A prospective observational study was conducted at Imperial College London by continuously collecting data for up to one hour from healthy volunteers. The study approval was granted by an institutional review board: Local Ethics Committee of Imperial College London (ICREC ref.: 18IC4358).
- A retrospective validation was carried out using previously collected data [22] from patients that had been referred for diagnosis of obstructive sleep apnea (OSA) (Trial registration number: NCT03544086).

### 2.2. Patient and Public Involvement

Members of the public were involved in prior formative usability evaluations (IEC 62366) that informed the design of the study.

### 2.3. Eligibility Criteria

For the prospective study, healthy adults (aged 18–70) were recruited. Subjects were excluded if they had any known cardiac or respiratory conditions, experienced difficulties breathing, were unwilling to follow the protocol of the study, or if they had hair on their neck. All subjects signed informed consent forms and were provided with oral and written explanations of the study protocol before starting.

The data used for retrospective validation were obtained in a study that included adults aged 18–70, excluding those who were not fluent in English or had special communication needs; those with a known allergy to adhesive dressings; subjects with physical or mental impairments, which would make them unable to use the new technology on their own; subjects with electronic body implants; and subjects with extremely lose skin in the neck area, which would make the device swing if the neck moved. Patients were recruited consecutively. Full details of this study can be found in our previously published paper [22].

### 2.4. Description of Test Device: AcuPebble RE100

**Figure 1:**
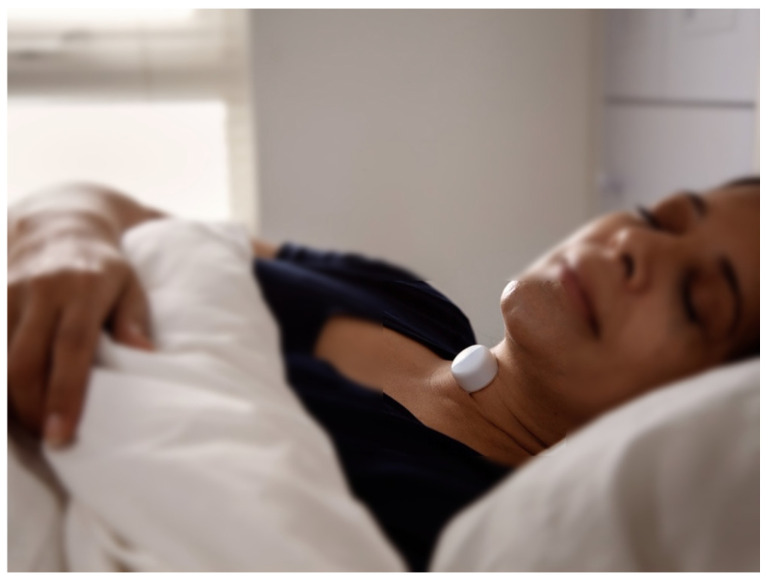
Model (not patient) wearing the sensor.

AcuPebble RE100 (Acurable, London, UK) is a device consisting of a CE-marked and FDA-approved wearable sensor (same sensor used in AcuPebble SA100 (Acurable, London, UK), which is intended for OSA diagnosis), along with a companion smartphone app that collects data wirelessly transmitted from the sensor, and analysis software platform that outputs both conventional validated physiological parameters and mathematical signal processing features, which can be used both for clinical and algorithm development research not specific to OSA diagnosis as in AcuPebble SA100 (Acurable, London, UK). The sensor measures 2.9 cm in diameter, 1.4 cm in height, and weighs 7 g, and it is able to run on battery for up to 20 h without charging. A double-sided medical-grade adhesive is used to attach the sensor to the neck anywhere between the laryngeal prominence of the thyroid cartilage and the supra-sternal notch [22]. A figure showing a model (not patient) wearing the sensor is shown in Figure 1.

### 2.5. Description of Reference Device (Prospective Study): Capnostream™35

A multi-parameter end-tidal CO_2_ capnography system, the Capnostream™35 (Medtronics, Minneapolis, MN, USA), was used as a ground truth reference for continuous RR measurements. This system was chosen based on it being an FDA-approved breathing frequency monitor [24] and its use in other research studies [25,26,27,28,29] and in clinical settings [30].

### 2.6. Description of Reference Device (Retrospective Validation): Embletta MPR Sleep System

A type-III cardiorespiratory polygraphy monitor, the Embletta MPR Sleep System (Natus Medical, Pleasanton, CA, USA), specifically the thoracic and abdominal piezoelectric respiratory bands, was used as a ground truth reference for the retrospective validation study.

### 2.7. Prospective Study Data Collection

After obtaining the participants’ signed consent form, basic demographic data including age, height, and weight were collected.

Subjects rested in a supine position, in a room with ambient noise intensity ranging between 35 and 40 dB. Each subject had the AcuPebble RE100 (Acurable, London, UK) device placed on their neck region. A nasal cannula and finger pulse oximeter (placed on the left index finger), connected to the bedside Capnostream™35 (Medtronic, Minneapolis, MN, USA), were then attached to the user. All sensors were handled and applied to the subject by a study organizer and cleansed with alcohol wipes in between subjects.

The complete study procedure can be found in Appendix A Table A1. The experiment was performed in one run, per subject, with the data collection protocol composed of two main phases:

1. Guided Breathing: In this phase, subjects followed a visual metronome to obtain paired measurements at a pre-defined rate. This phase represented a no-artifact condition.
2. With Artifacts: In this phase, the subjects would no longer follow the visual metronome. They breathed normally while artificial noise was applied through speakers, or performed tasks to mimic some possible physiological artifacts during sleep, such as coughing, groaning, and head movements.

#### 2.7.1. Guided Breathing

Table 1 outlines the breathing cadences followed for this section of the experiment. This phase started with the visual metronome pulsating at a rate representing 14 BPM for two minutes. A study organizer observed the subjects and ensured that they were following the visual metronome correctly. The controlled breathing phase then started by gradually changing the visual metronome’s rate from 14 BPM to 4 BPM, with a 2 BPM reduction every minute. Next, the visual metronome setting gradually changed from 14 BPM to 30 BPM, increasing by 2 BPM every minute. Afterwards, the subjects were instructed to perform shallow breaths, with the visual metronome set to 24 BPM for two minutes. Next, the visual metronome would change abruptly at larger increments, reducing from 14 BPM to 9 BPM, 6 BPM, and 4 BPM, and then increasing from 14 BPM to 19 BPM, 22 BPM, 24 BPM, and lastly 27 BPM. During this phase of abrupt changes, each rate would last for two minutes. Table 1 outlines the details of this process.

#### 2.7.2. With Artifacts

Table 2 outlines the induced artifacts and intervals for this phase of the experiment. This phase started with physiological noise, where the subjects were asked to perform groaning, snoring, and coughing actions. Afterwards, environmental noise at 65 dBA, with and without background talking, was introduced. All environmental noise was projected with pre-recorded audio clips and mimicked hospital noises. Lastly, the subjects performed head movements, requiring them to tilt their head up and down, left to right, and then shake their head left and right once per verbal cue.

### 2.8. Sample Size

The sample size was estimated based on the Root Mean Squared Deviation (RMSD) between AcuPebble RE100 (Acurable, London, UK)’s breathing rate output and the Capnostream™35 (Medtronic, Minneapolis, MN, USA) being less than 3 BPM across all the collected data points. Three BPM was chosen due to it being the maximum threshold for error included in many FDA 510(k) applications for breathing frequency monitors, as shown in Appendix A Table A2. To estimate the required sample size, a two-sided paired t-test power calculation was performed. This approach was chosen to determine the number of paired samples and subjects necessary to detect a statistically significant difference between the two devices. The calculation aimed for a significance level of 5% (α = 0.05) and 80% statistical power (1 − β = 0.80) and considered the expected effect size based on preliminary data. Based on this power analysis, it was concluded that a total of 14 subjects, with 14,307 paired samples, would be required for the study.

### 2.9. Statistical Analysis

The statistical analysis was heavily influenced by metrics recorded in FDA submissions for breathing frequency monitors, as shown in Appendix A Table A1 and Table A2. Furthermore, metrics included in the ISO80601-2-71 standard were used due to their relevance in showing equivalency for continuous monitors and their use in other validation studies [31].

These metrics include RMSD, mean error, and standard deviation (SD) of the difference between paired measurements throughout the study, as described by the protocol where the presence of different physiological, physical, and environmental noises was taken into account, enabling the evaluation of their effects on the measurement accuracy. Agreement between devices was further studied using Bland–Altman analysis [32]. Metrics associated with the Bland–Altman plot were included, such as bias (mean difference) and limits of agreement (LOAs), indicating the 95% bounds of the difference between paired measurements of both methods. The components of variance technique was used to account for inter-subject variance and to address longitudinal correlation affecting the SD and LOAs [33]. Linear correlation of observations between the test and reference devices with a 95% confidence interval were shown in scatter plots. Plotted data on the scatter and Bland–Altman plots were assigned a size and transparency factor to visually interpret density of the data over different ranges.

The above metrics were calculated for the entire dataset for both the prospective and retrospective validations. In the case of the prospective study, the metrics were also calculated for separate segmented events shown in Table 1 and Table 2, including controlled breathing, abrupt breathing, shallow breathing, physiological noises, environmental noises, and physical movements. The guided breathing section was further segmented into tachypnea (>20 BPM), normal breathing (10–20 BPM), and bradypnea (<10 BPM) to understand bias at different RR ranges.

Based on similar studies [34,35], the accuracy rate was calculated as the percentage of time AcuPebble RE100 (Acurable, London, UK)’s breathing rate output was within ±2 BPM of the reference device. Furthermore, the percentage of time AcuPebble RE100 (Acurable, London, UK) was able to provide an output for RR was also measured. All designated “rest periods” as shown in the study protocol (Appendix A Table A1) were removed from the analysis. All analyses were performed using Matlab Version R2022.

## 3. Results

### 3.1. Participants

**Figure 2:**
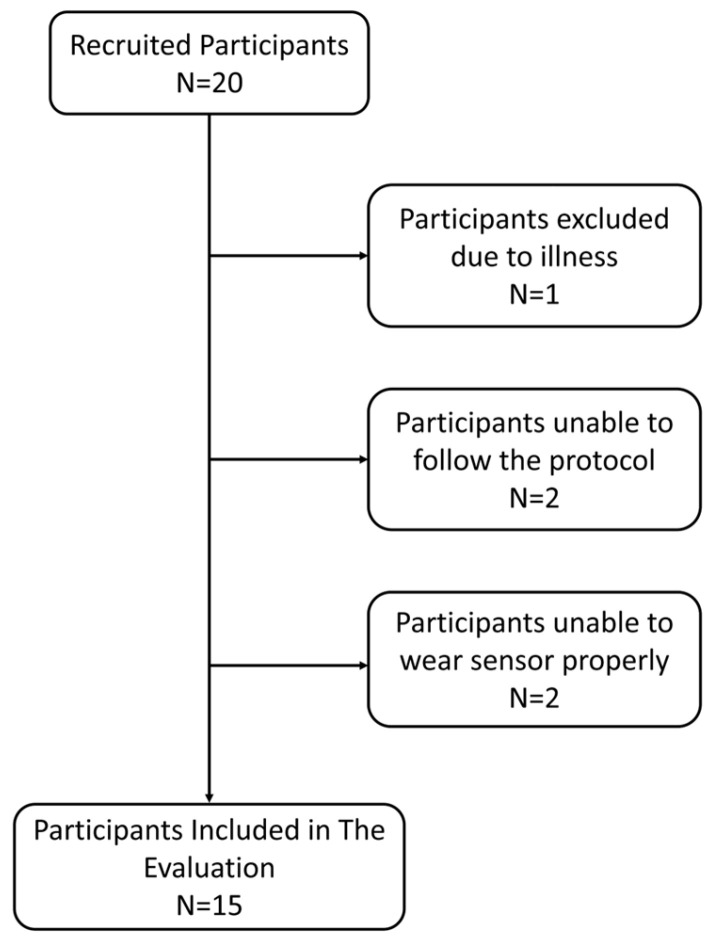
Flow of participants and data sufficiency diagram.

In the controlled experiment for the prospective study, twenty subjects were recruited over a 2-month duration between September 2021 and November 2021. Five subjects were excluded from the study due to two subjects having hair on the neck location of the sensor (the possibility of shaving was not included in the protocol), two being unable to follow the study, and one being acutely ill. All the subjects included were healthy and claimed not to have any known respiratory or cardiac irregularities. The demographic data of the participants included are shown in Table 3, and a flow diagram of the participants is shown in Figure 2.

The database corresponding to the clinical study described in [22] contained signals from 150 consecutive patients with demographic characteristics, as shown in Table 4.

### 3.2. Respiratory Rate Accuracy in Controlled Experiment (Prospective Study)

A total of 1.3% of the data points were excluded from the analysis either due to the Capnostream™35 (Medtronic, Minneapolis, MN, USA) not outputting the data due to the drift in the location of the nasal cannula or experimental disruptions during the no-motion conditions. In total, 16,872 paired data points were collected.

The RR estimation results over the complete dataset are shown in Table 5, with the overall error, bias, and RMSD equal to 1.83 ± 2.09, 0.63 ± 2.71, and 2.78 BPM, respectively. The percentage of data points within ±2 BPM from the reference device measurements was equal to 78.86 ± 17.36% out of the 95.63 ± 6.62% values outputted.

**Figure 3:**
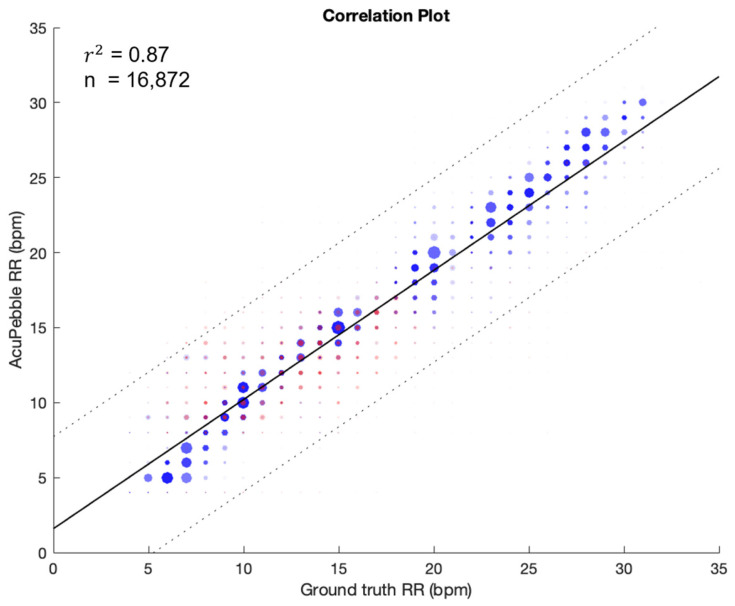
Linear regression of the complete prospective study dataset, showing comparison between the test and reference devices during guided breathing (blue) and artifacts (red).

**Figure 4:**
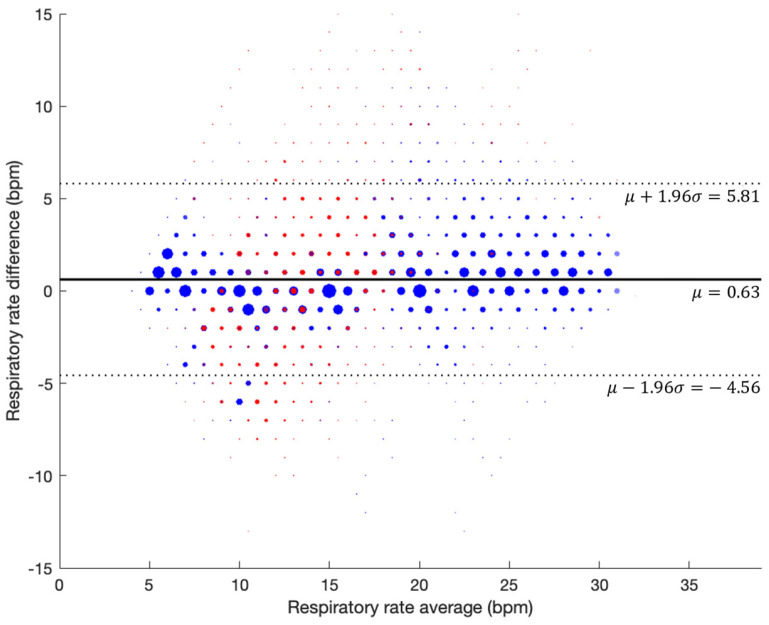
Bland–Altman plot showing agreement between the test and reference devices for the complete prospective study, with artifacts shown in red.

Figure 3 outlines the scatter plot showing the agreement between the reference and test devices for the complete dataset, with guided breathing (blue) and artifacts (red) sections, reporting an r^2^ value of 0.87. Figure 4 shows the Bland–Altman plot where a bias of 0.63 BPM was obtained, with the upper and lower LOAs being 5.81 BPM and −4.56 BPM, respectively. Furthermore, Table 6 shows the percentages of the samples with values within different ranges over the guided breathing phase and the complete data.

#### 3.2.1. Guided Breathing (No Artifacts)

The guided part of the experiment covered a reference RR range from 4 to 30 BPM. As shown in Table 7, the controlled breathing phases had a smaller margin of error (1.21 BPM ± 1.36 BPM) compared to the shallow breathing (2.00 BPM ± 2.06 BPM) and abrupt changes (1.66 BPM ± 1.83 BPM), with all having RMSD values less than 3 BPM. When analyzing these segments by the RR range, tachypnoea suffered from a slightly higher bias (1.20 BPM). In Table 8, it is shown that values were outputted 100% of the time during all the scenarios of guided breathing. Furthermore, as shown in Table 7, when segmenting the data into breathing rates, normal breathing and bradypnea had very high accuracy rates, with tachypnoea being slightly lower. The results from the entire guided breathing section yielded a mean error of 1.48 BPM ± 1.67 BPM with an RMSD less than 3 BPM.

**Figure 5:**
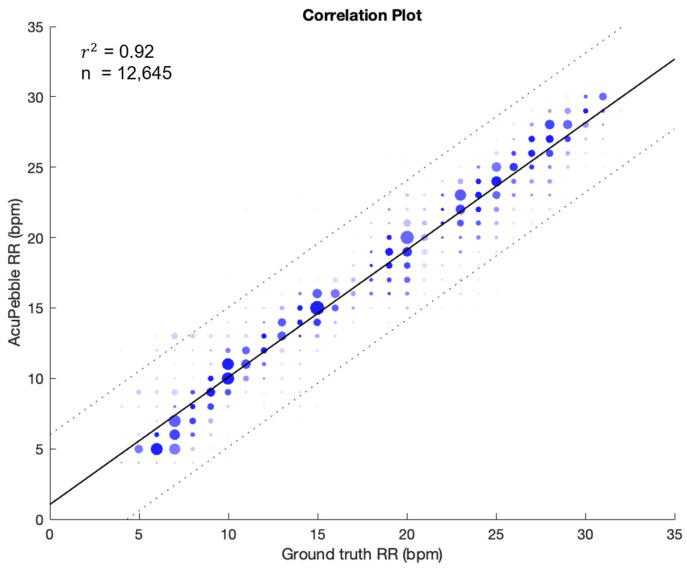
Linear regression of the guided breathing phase of the prospective study showing comparison between the test and reference devices.

**Figure 6:**
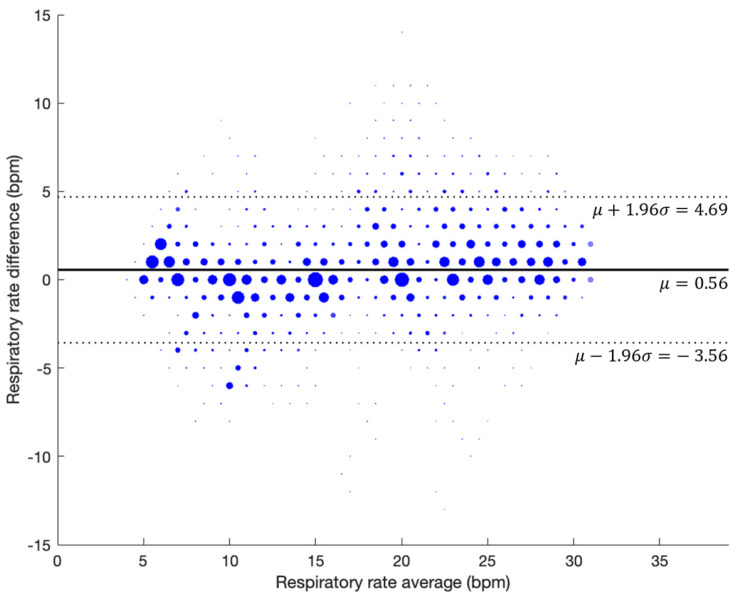
Bland–Altman plot showing agreement between the test and reference devices for the guided breathing phase of the prospective study.

Figure 5 outlines the scatter plot showing the agreement between AcuPebble RE100 (Acurable, London, UK) and the reference device during guided breathing. With 12,645 paired measurements, the coefficient of determination (r^2^) was 0.92, and the RMSD was 2.23 BPM. Figure 6 shows the Bland–Altman plot. A bias of 0.56 BPM was obtained, with the upper and lower limits of agreement (LOAs) being 4.69 BPM and −3.56 BPM, respectively.

#### 3.2.2. With Artifacts

As shown in Table 9, a higher error was evident when artifacts were introduced compared to the no-motion situations. Of the physiological noise, snoring evidently was not as error-prone (1.64 BPM ± 1.70 BPM) as groaning (2.88 BPM ± 2.98 BPM) or coughing events (2.59 BPM ± 2.15 BPM), being the only artifacts with RMSD values less than 3 BPM. Artificial noise had an increased positive bias, thus overestimating RR compared to the reference system. Head movement noise appeared to affect the results similarly to artificial noise. Out of the artifacts, artificial noise had the lowest number of values outputted, while the other artifacts are all close to 100%, as shown in Table 10.

### 3.3. Evaluation During Natural Sleep (Retrospective Study)

The reference system used for the evaluation during natural sleep was a type-III cardiorespiratory polygraphy monitor: the Embletta MPR Sleep System (Natus Medical, Pleasanton, CA, USA). The evaluation of the RR output of AcuPebble RE100 (part of the AcuPebble SA100 system) (Acurable, London, UK) during natural sleep was carried out counting the respiratory oscillations of the chest and abdomen effort bands of the Embletta system. A 60 s window was used to calculate the RR for each two-second period of the signal.

For each patient, a 30 min period was selected where the comparison was completed. This period was nominally defined as starting two hours after the patient initiated the recording. In cases in which the counting of the peaks from effort bands (i.e., the gold-standard) could not be carried out due to sensor misplacement or signal artifacts in the Embletta bands, which made it impossible to identify the peaks visually, the comparison window was moved by 10 min. The 10 min comparison window movement was repeated until a complete 30 min segment without visible Embletta sensor failure was found.

No adjustment was completed based on the signal quality of AcuPebble RE100 (Acurable, London, UK). One patient was eliminated from the comparison as there was no continuous period of 30 min where a reliable breathing rate could be computed from the Embletta signals.

Once a valid 30 min comparison period was found, the breathing rate of AcuPebble RE100 (Acurable, London, UK) was compared to that of the Embletta every 10 s.

The overall performance of AcuPebble RE100 (Acurable, London, UK) when evaluated during natural sleep is shown in Table 11. The comparison of a total of 26,820 data points yielded a mean error of 1.40 BPM ± 1.11 BPM, also with RMSD less than 3 BPM, comparable to the overall performance during the controlled experiment above. Table 12 lists the percentages of samples in the complete dataset with values within a certain range.

**Figure 7:**
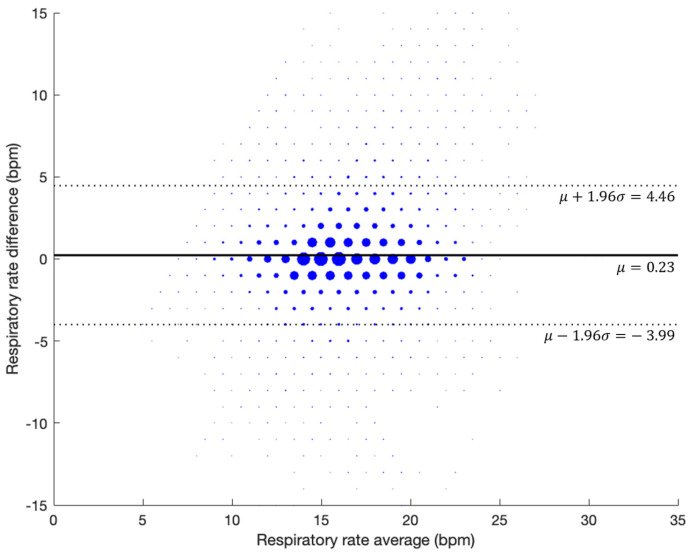
Bland–Altman plot showing the agreement for all the subjects in the retrospective validation.

The combined Bland–Altman plot corrected for repeated measurements per subject for all the data, together with the bias and standard deviation, is shown in Figure 7. The bias obtained was −0.23, with the upper and lower LOA being 4.46 BPM and −3.99 BPM, respectively, while the RMSD was 2.46 BPM.

## 4. Discussion

This study evaluated AcuPebble RE100 (Acurable, London, UK)’s ability to accurately define RR compared to a reference end-tidal CO_2_ capnography system and a type-III cardiorespiratory polygraphy monitor. For the prospective study, AcuPebble RE100 (Acurable, London, UK)’s algorithm was tested during no artifact to assess its ability to output RR over a broad range (4–30 BPM) with both consistent and abrupt changes in breathing, and with additional artifacts, including physiological noises and movements, which may occur during sleep, to assess its robustness. The algorithm was also assessed with the data obtained during natural sleep for a retrospective validation. The results in this study demonstrate AcuPebble RE100’s reliability in breathing frequency monitoring for sleep applications, within a clinically relevant margin of error, producing an overall absolute RMSD under 3 BPM for both the controlled experiment and evaluation during natural sleep.

Our results in the prospective observational study are comparable to a study with a similar study design, assessing an FDA-approved acoustic respiration monitor (Radical-7, Masimo, Irvine, CA, USA) [36] against a reference capnograph (Capnostream™20, Medtronic, Minneapolis, MN, USA) [35]. AcuPebble RE100 (Acurable, London, UK) had a lower mean error throughout no-motion (guided breathing) conditions, with the errors for controlled breathing being 1.21 BPM ± 1.36 BPM and abrupt breathing 1.66 BPM ± 1.83 BPM compared to the results of 1.62 BPM ± 0.62 BPM and 2.19 BPM ± 0.84 BPM, respectively. In addition, AcuPebble RE100 (Acurable, London, UK) was able to output an improved accuracy rate within ±2 BPM during controlled and abrupt breathing with 90.60 ± 10.06% and 86.16 ± 9.94% as opposed to 76.3 ± 13% and 63.0 ± 15.7%, respectively. Furthermore, AcuPebble RE100 (Acurable, London, UK)’s error did not increase significantly during tachypnoea, as was observed in their study. However, a slight drop in the agreement with the reference device was observed during tachypnoea and shallow breathing, which can be explained by the relatively weak signal and the irregularities that it suffered during these phases, which makes RR estimation more challenging. Another reason could be the difficulty in executing these breathing phases, which might have caused the participants to struggle to follow the prompt, especially in high breathing rates. Nonetheless, it is important to note that the results are still within the acceptable range of error. AcuPebble RE100 (Acurable, London, UK) provided measurements during a much higher percentage of time with respect to the comparable Radical-7 (Masimo, Irvine, CA, USA) study during physiological noises, with a ratio of 98.41 ± 6.15% compared to 58.0 ± 14.8%. During head movements, AcuPebble RE100 (Acurable, London, UK) provided measurements 100% of the time compared to their percentage, which was 71.1% of the time. With regard to environmental noise artifacts, the Radical-7 study did not suggest the accuracy of the results when outputting the data. In contrast, AcuPebble RE100 (Acurable, London, UK) suffered when subjected to high environmental noises. Given the typical sleep conditions, it is unlikely that AcuPebble RE100 (Acurable, London, UK) would be subject to noises above 65 dBA for prolonged periods of time, as was the case in this study, especially as the WHO recommends the noise levels in hospitals to be below 35 dBA, and this would already be significantly higher than in a normal domestic environment.

The performance of AcuPebble RE100 (Acurable, London, UK) when assessed during natural sleep in the retrospective validation also shows comparable results to the prospective study above, with an overall RMSD of 2.04 BPM ± 1.38 BPM and mean error of 1.40 BPM ± 1.11 BPM, while the output percentage of the time within ±2 BPM accuracy was 84.29%. These results are promising given that the proposed device from the comparable study (Radical-7, Masimo, Irvine, CA, USA) is an FDA-approved breathing frequency monitor that is suitable for both motion (with artifacts) and no-motion (with no artifacts) conditions that demonstrated a reduced percentage of time providing measurements during motion compared to AcuPebble RE100 (Acurable, London, UK), while AcuPebble RE100 (Acurable, London, UK) was able to outperform Radical-7’s measurement accuracy in both controlled and abrupt breathing. Our results are also comparable with another FDA-approved device recently included in the National Institute for Health and Care Excellence (NICE) advice on Medtech innovation briefing (RespiraSense, PMD Solutions, Cork, Ireland) [37,38]. During no-motion conditions, both AcuPebble RE100 (Acurable, London, UK) and RespiraSense achieved biases of 0.56 BPM and 0.38 BPM, respectively, both under ± 1 BPM. RespiraSense was also tested under the influence of motion artifact, achieving a bias of −1.72 BPM compared to AcuPebble RE100 (Acurable, London, UK), which achieved a lower bias of 1.05 BPM. However, it is important to note that the motion artifacts introduced were different in the study evaluating RespiraSense due to differences in the sensing methodology of the two devices. Furthermore, only 62 data points were used for the comparison of RespiraSense (PMD Solutions, Cork, Ireland) against capnography as compared to the total of 16,872 data points in the prospective evaluation of AcuPebble RE100 (Acurable, London, UK).

In the context of sleep studies, AcuPebble RE100 (Acurable, London, UK) provides a user-friendly alternative to quantify RR, thus increasing the scope and accessibility for remote overnight monitoring. Paired with its ability to detect apnea, it could provide clinicians with data to identify changes in breathing patterns during sleep, calculating metrics including Respiratory Rate Variability, which are believed to be under-studied due to a lack of user-friendly tools [39,40]. Lastly, the clinical benefits of continuous RR monitoring are still developing, with the current applications including detecting abnormal RR (below 6 or greater than 24) and using it to aid early warning scores for detecting deterioration in patients [41]. Therefore, there are benefits in providing a user-friendly and non-invasive option for continuous monitoring to conduct exploratory research and expand the scope of RR monitoring.

## 5. Limitations

The controlled nature of the study compared with capnography poses certain limitations by default. Firstly, only healthy participants with no known respiratory issues were recruited. Furthermore, by using a visual metronome, the subjects may have been subject to unnatural breathing cycles, which may not be indicative of spontaneous breathing. The physiological noises were also mimicked and studied in frequencies likely higher than in most sleep applications. Therefore, the error shown in the results for these segments may seem to be more significant compared to that of a real-sleep study.

However, the above aspects are somewhat redeemed by the retrospective study including patients that had been referred for OSA diagnosis since they had a variety of comorbidities and were sleeping in their natural uncontrolled environments.

A possible direction for future work would be to include physiological parameters such as physical activity level and lung function measures in order to provide more interpretations of the results, and, with sufficient data in each category, a thorough sub-group analysis of the capabilities of the system would be achievable.

## 6. Conclusions

Overall, the performance of the RR monitoring capability of AcuPebble RE100 (Acurable, London, UK) was assessed both under a controlled condition and during natural sleep, with and without artifacts. This study provides evidence that AcuPebble RE100 (Acurable, London, UK), a user-friendly and minimally invasive wearable device, is highly effective for overnight RR monitoring. The device demonstrated excellent accuracy, with the measurements consistently falling within the clinically relevant margins of error. Further analysis revealed that, even in the presence of physiological, environmental, and physical artifacts, the device generally maintained its performance, which is a critical factor for reliable overnight monitoring.

Moreover, the ease of use and comfort provided by the AcuPebble RE100 (Acurable, London, UK) device are significant in making it suitable for real-world, long-term monitoring applications, particularly in home settings or during sleep studies. Compared to more traditional clinical methods like capnography, which often require bulky equipment or specific clinical environments, AcuPebble RE100 (Acurable, London, UK) offers a practical solution for continuous RR assessment, being a more accessible, accurate, and patient-friendly RR monitoring system.

## Figures and Tables

**Table 1:** Guided breathing steps and time intervals. Between each breathing step, the visual metronome would guide the user to a “resting rate” of 14 BPM before starting the next section.

Breathing Step	BPM	Minute	Breathing Step	BPM	Minute
Controlled Breathing	14	1 min	Shallow Breathing	24	2 min
	12				
	10				
	8				
	6				
	4				
	14 (Rest)				
	16		Abrupt Changes	14	2 min
	18			9	
	20			6	
	22			4	
	24			14	
	26			19	
	28			24	
	30			27	

**Table 2:** List of artifacts used in this study, including time interval.

Artifact Type	Event	Rate	Minute
Physiological Noise	Groaning	Constant	2 min
	Snoring	Constant	
	Coughing	10–12 Coughs	
Environmental Noise	65 dBA Hospital Noises	Constant	2 min
	65 dBA Hospital Noises + talking		
Physical Noises (Head movement)	Lateral head movements	Transition every 10 s	1 min
	Posterior head movements		
	Lateral head shake		

**Table 3:** Characteristics of the 15 participants of the prospective RR validation study.

Characteristic	Measure	Value
Age (years)	Median	26
	Mean	29
	Standard deviation	6
	Range	[24, 47]
BMI	Median	23.5
	Mean	23.4
	Standard deviation	3.6
	Range	[19.4, 33.8]
Weight (kg)	Median	74
	Mean	71.3
	Standard deviation	15.3
	Range	[54, 100]
Height (cm)	Median	172
	Mean	173.7
	Standard deviation	11.4
	Range	[153, 193]
Number of participants per BMI classification	Underweight (<18.5)	0 (0%)
	Healthy weight (18.5–24.9)	13 (86.67%)
	Overweight (25–29.9)	1 (6.67%)
	Obese (30–39.9)	1 (6.67%)
	Severely obese (>40)	0 (0%)
Sex	Male	10 (66.7%)
	Female	5 (33.3%)

**Table 4:** Characteristics of the 150 patients in [22] corresponding to the database used for retrospective evaluation of the RR algorithm.

Characteristic	Measure	Value
Age (years)	Median	45
	Mean	44
	Standard deviation	11
	Range	[21, 65]
BMI Self-reported. Data available from 128 patients (84.2%)	Median	29.9
	Mean	31.2
	Standard deviation	7.6
	Range	[17.6, 56.6]
Weight (kg) Self-Reported. Data available from 129 patients (84.9%)	Median	92
	Mean	95.3
	Standard deviation	25.7
	Range	[45.7, 190]
Height (cm) Self-reported. Data available from 132 patients (86.8%)	Median	175.2
	Mean	174.4
	Standard deviation	9.8
	Range	[150, 197]
Number of patients per BMI classification	Underweight (<18.5)	1 (0.7%)
	Healthy weight (18.5–24.9)	26 (17.3%)
	Overweight (25–29.9)	36 (24%)
	Obese (30–39.9)	51 (34%)
	Severely obese (>40)	12 (8%)
Sex	Male	107 (71.3%)
	Female	43 (28.7%)
Ethnicity (Number of patients)	White British	47 (31%)
	White other	19 (12.67%)
	Asian or Asian British (excluding the ones below)	31 (20.67%)
	Black or Black British (excluding the ones below)	3 (2%)
	Indian	2 (1.33%)
	Pakistani	2 (1.33%)
	White or Black African	2 (1.33%)
	Chinese	1 (0.67%)
	White or Black Caribbean	5 (3.33%)
	Other	38 (25.34%)
Most common comorbidities	High blood pressure	38 (25.3%)
	Diabetes	17 (11.3%)
	Asthma	10 (6.7%)

**Table 5:** Overall RR metrics of the prospective study listing the mean error, bias, RMSD, and data points for each segment, in addition to the percentages of time the reference and test devices were within ±2 BPM, and the percentages of values outputted.

Segmented RR	Error (mean ± std BPM)	Bias (mean ± std BPM)	RMSD (BPM)	Data Points	In Range (mean ± std %)	Value Outputted (mean ± std %)
Complete Data	1.83 ± 2.09	0.63 ± 2.71	2.78	16,872	78.86 ± 17.36	95.63 ± 6.62

**Table 6:** Percentages of samples for all subjects with values within different ranges in the prospective study.

Phases	≤±1 bpm	≤±2 bpm	≤±3 bpm	≤±4 bpm	≤±5 bpm
Guided Breathing	75.01%	87.39%	92.64%	95.96%	97.70%
Complete Data	71.79%	84.03%	89.66%	93.45%	95.79%

**Table 7:** Guided breathing metrics of RR indicating mean error, bias, RMSD, and data points for each segment. Data from controlled breathing, shallow breathing, and abrupt breathing were then segmented into tachypnoea, bradypnea, and normal breathing.

Segmented RR	Error (BPM)	Bias (BPM)	RMSD (BPM)	Data Points
Controlled Breathing	1.21 ± 1.36	0.59 ± 1.71	1.81	5653
Shallow Breathing	2.00 ± 2.06	1.40 ± 2.52	2.88	835
Abrupt Changes	1.66 ± 1.83	0.42 ± 2.43	2.46	6157
Tachypnoea	1.83 ± 1.93	1.20 ± 2.37	2.66	4271
Normal	1.18 ± 1.39	0.45 ± 1.77	1.83	4524
Bradypnea	1.43 ± 2.14	−0.02 ± 2.1	2.14	3850
Guided Breathing	1.48 ± 1.67	0.56 ± 2.16	2.23	12,645

**Table 8:** Tabulated results for the guided breathing segments indicating the percentages of time the reference and test device were within ±2 BPM.

Segmented RR	In Range (mean ± std %)	Value Outputted (mean ± std %)
Controlled Breathing	90.60 ± 10.06	100 ± 0
Shallow Breathing	74.17 ± 21.90	100 ± 0
Abrupt Changes	86.16 ± 9.94	100 ± 0
Tachypnoea	79.36 ± 13.74	100 ± 0
Normal	93.89 ± 6.78	100 ± 0
Bradypnea	90.37 ± 7.79	100 ± 0
Guided Breathing	87.35 ± 7.71	100 ± 0

**Table 9:** Results of RR segmented into the various artifacts in the prospective study.

Segmented RR	Error (mean ± std BPM)	Bias (mean ± std BPM)	RMSD (BPM)	Data Points
Physiological Noise	2.35 ± 2.40	0.25 ± 3.35	3.36	2130
- Groaning	2.88 ± 2.98	0.92 ± 4.04	4.14	718
- Snoring	1.64 ± 1.70	−0.36 ± 2.33	2.36	742
- Coughing	2.59 ± 2.15	0.19 ± 3.36	3.37	670
Artificial Noise	3.33 ± 2.78	1.69 ± 4.00	4.34	1156
Movement Noise	3.54 ± 3.27	1.05 ± 4.71	4.82	941

**Table 10:** Percentages of time the reference and test device were within ±2 BPM during artifacts in the prospective study.

Segmented RR	In Range (mean ± std %)	Value Outputted (mean ± std %)
Physiological Noise	81.88 ± 14.88	98.41 ± 6.15
- Groaning	70.56 ± 33.92	95.33 ± 18.07
- Snoring	93.60 ± 11.42	100 ± 0
- Coughing	77.63 ± 26.59	100 ± 0
Artificial Noise	68.50 ± 24.08	75.36 ± 33.58
Movement Noise	71.85 ± 23.31	100 ± 0

**Table 11:** Mean error, bias, RMSD, and data points for the retrospective validation study.

Error (BPM)	Bias (BPM)	RMSD (BPM)	Data Points
1.40 ± 1.11	−0.23 ± 1.87	2.46	26,820

**Table 12:** Percentages of samples for all subjects with values within different ranges in the retrospective validation study.

≤±1 bpm	≤±2 bpm	≤±3 bpm	≤±4 bpm	≤±5 bpm
72.38%	84.29%	90.11%	93.50%	95.56%

## Data Availability

Due to restrictions in the ethics document, data beyond those presented in this publication cannot be distributed. If you have any questions, please email the corresponding author (e.rodriguez@imperial.ac.uk).

## Appendix A

**Table A1:** Study protocol.

Phase	Step	Time (min)	Respiratory Rate (BPM)	Action
Experiment Start (Phase 1)	Breathing Normally	1	Variable	Unspecified
Phase 2 (Controlled Breathing)	Breath at pace	1	14	Unspecified
Phase 3 (Controlled Breathing)	Breath at pace	1	12	Unspecified
Phase 4 (Controlled Breathing)	Breath at pace	1	10	Unspecified
Phase 5 (Controlled Breathing)	Breath at pace	1	8	Unspecified
Phase 6 (Controlled Breathing)	Breath at pace	1	6	Unspecified
Phase 7 (Controlled Breathing)	Breath at pace	1	4	Unspecified
Phase 8 (Controlled Breathing)	Breath at pace	2	14	Unspecified
Phase 9 (Controlled Breathing)	Breath at pace	1	16	Unspecified
Phase 10 (Controlled Breathing)	Breath at pace	1	18	Unspecified
Phase 11 (Controlled Breathing)	Breath at pace	1	20	Unspecified
Phase 12 (Controlled Breathing)	Breath at pace	1	22	Unspecified
Phase 13 (Controlled Breathing)	Breath at pace	1	24	Unspecified
Phase 14 (Controlled Breathing)	Breath at pace	1	26	Unspecified
Phase 15 (Controlled Breathing)	Breath at pace	1	28	Unspecified
Phase 16 (Controlled Breathing)	Breath at pace	1	30	Unspecified
Transition to Rest	Breath at pace	1	19	Unspecified
Rest	Breath at pace	1	variable	Unspecified
Phase 17 (Shallow Breathing)	Shallow Breathing	2	24	Unspecified
Transition to Rest	Breath at pace	1	18	Unspecified
Rest	Breath at pace	1	14	Unspecified
Phase 19 (Abrupt Changes)	Abrupt Changes	2	9	Unspecified
Phase 20 (Abrupt Changes)	Abrupt Changes	2	6	Unspecified
Phase 21 (Abrupt Changes)	Abrupt Changes	2	4	Unspecified
Phase 22 (Abrupt Changes)	Abrupt Changes	2	14	Unspecified
Phase 23 (Abrupt Changes)	Abrupt Changes	2	19	Unspecified
Phase 24 (Abrupt Changes)	Abrupt Changes	2	22	Unspecified
Phase 25 (Abrupt Changes)	Abrupt Changes	2	27	Unspecified
Transition to Rest	Breath at pace	1	18	Unspecified
Rest	Breath at pace	1	14	Unspecified
Phase 26 (Physical Noises)	Groaning Events	2	Variable	Groan Event
Rest	Breath at pace	1	Variable	Unspecified
Phase 27 (Physical Noises)	Snoring Event	2	Variable	Snoring Event
Rest	Breath at pace	1	Variable	Unspecified
Phase 28 (Physical Noises)	Cough Event	2	Variable	Cough Event
Rest	Breath at pace	1	Variable	Unspecified
Phase 29 (Natural Noise)	Hospital Noises (65 dB)	2	Variable	Unspecified
Rest	Breath at pace	1	Variable	Unspecified
Phase 30 (Natural Noise)	Talking Noises (65 dB)	2	Variable	Unspecified
Rest	Breath at pace	1	14–16 bpm	Unspecified
Phase 31 (Physical Movement)	Move head right and left	1	Variable	Change every 10 s
Phase 32 (Physical Movement)	Rest	1	Variable	Unspecified
Phase 33 (Physical Movement)	Move Head Up and center	1	Variable	Change every 10 s
Phase 34 (Physical Movement)	Rest	1	Variable	Unspecified
Phase 35 (Physical Movement)	Move head: right–left then back to center	1	Variable	Change every 10 s
Phase 36 (Physical Movement)	Rest	1	Variable	Unspecified
Exit	(1) Export iPad data to Box (2) Save montage on lab laptop from the SOMNO		Unspecified	Unspecified

**Table A2:** FDA-approved breathing frequency monitors.

Company Name [URL]	Primary Predicate Device [Technology]	Accuracy (Range)	Intended Use	Year
Biobeat Technologies Ltd. (Petah Tikva, Israel) [https://www.accessdata.fda.gov/cdrh_docs/pdf21/K212153.pdf accessed on 22 November 2024]	BB-613WP Patch [Wrist PPG]	±3 BPM (4–40 BPM)	Spot measurements Intended for adults in clinical and home settings Not intended for critical care patients	2022
Sound Life Sciences, Inc. (Seattle, WA, USA) [https://www.accessdata.fda.gov/cdrh_docs/pdf21/K211387.pdf accessed on 22 November 2024]	Breathe Easy Mobile Respiratory Monitor [Phone microphone]	Not Included	Spot measurements Intended for adults in clinical and home settings Not a vital sign or apnea monitor Not used on patients with uncontrolled limb movement	2021
Masimo Corporation (Irvine, CA, USA) [https://www.accessdata.fda.gov/cdrh_docs/pdf19/K191882.pdf accessed on 22 November 2024]	Masimo Root Monitoring System and Accessories (K171121) [PPG]	±3 BPM (8–35 BPM)	Continuous monitoring Intended for adults and pediatrics in clinical settings Only stationary conditions	2020
Philips Healthcare (Best, The Netherlands) [https://www.accessdata.fda.gov/cdrh_docs/pdf19/K192875.pdf accessed on 22 November 2024]	Philips Wearable Biosensor-G5 [ECG and accelerometer]	±3 BPM (3–40 BPM)	Continuous monitoring Intended for adults in clinical settings	2020
Xandar Kardian Inc. (Toronto, ON, Canada) [https://www.accessdata.fda.gov/cdrh_docs/pdf20/K202464.pdf accessed on 22 November 2024]	Vital Sign Monitoring Sensor [Radar]	Not Included	Continuous monitoring Intended for adult patients in clinical and home settings Not meant for acute treatment	2021
Nanowear Inc. (Brooklyn, NY, USA) [https://www.accessdata.fda.gov/cdrh_docs/pdf21/K212160.pdf accessed on 22 November 2024]	SimpleSENSE Platform [Thoracic impedance]	±2 BPM (6–22 BPM)	Intended for adult patients in clinical and home settings Not intended for critical care patients Only stationary conditions	2020
Masimo Corporation (Irvine, CA, USA) [https://www.accessdata.fda.gov/cdrh_docs/pdf19/K193242.pdf accessed on 22 November 2024]	Masimo Radical-7 Pulse Co-Oximeter and Accessories (K193242) [PPG]	±3 BPM (4–70 BPM)	Spot or continuous monitoring Intended for adult patients in clinical and home settings For adults, pediatrics, infants, and neonate patients	2020
Spire Health (San Francisco, CA, USA) [https://www.accessdata.fda.gov/cdrh_docs/pdf19/K192952.pdf accessed on 22 November 2024]	Spire Health Remote Patient Monitoring System [Force Sensor]	Not Included	Continuous monitoring Intended for adult patients in clinical and home settings Not intended for ICU or acutely ill cardiac patients	2020
Circadia Technologies Ltd. (Los Angeles, CA, USA) [https://www.accessdata.fda.gov/cdrh_docs/pdf20/K200445.pdf accessed on 22 November 2024]	The Circadia C100 System [Radar]	±2 BPM (7–38 BPM)	Spot or continuous monitoring Intended for adult patients in clinical and home settings Only stationary conditions	2020
Shenzhen Fiber Medical Technology Co., Ltd. (Shenzhen, China) [https://www.accessdata.fda.gov/cdrh_docs/pdf19/K190775.pdf accessed on 22 November 2024]	RHEA Vital Sign Vigilance System [Optical Fiber]	±2 BPM (7–45 BPM)	Continuous monitoring Intended for adult patients in clinical and home settings Only stationary conditions	2019
Masimo Corporation (Irvine, CA, USA) [https://www.accessdata.fda.gov/cdrh_docs/pdf18/K181956.pdf accessed on 22 November 2024]	Masimo MightySat Rx Fingertip Pulse Oximeter [Fingertip PPG]	±3 BPM (4–70 BPM)	Continuous monitoring Intended for adult patients in home environment Only stationary conditions	2018
VitalConnect Inc. (San Jose, CA, USA) [https://www.accessdata.fda.gov/cdrh_docs/pdf19/K192757.pdf accessed on 22 November 2024]	VitalConnect Platform, VitalPatch Biosensor [ECG and Accelerometer]	NA (10–30 BPM)	Continuous monitoring Intended for adult patients in clinical (excluding critical care) and home settings Only stationary conditions	2019
Snap40 Ltd. (Edinburgh, UK) [https://www.accessdata.fda.gov/cdrh_docs/pdf18/K182543.pdf accessed on 22 November 2024]	Wearable Vital Signs Monitoring System - Snap40 [Accelerometer, Gyroscope and oximeter]	NA (6–60 BPM)	Intermittent or spot measurements Intended for adult patients in clinical (excluding critical care) and home settings	2019
Covidien (Dublin, Ireland) acquired by Medtronic (Minneapolis, MN, USA) [https://www.accessdata.fda.gov/cdrh_docs/pdf14/K141518.pdf accessed on 22 November 2024]	Nellcor™ Bedside Respiratory Patient Monitoring System [PPG]	±1 BPM (4–40 BPM)	Continuous monitoring Intended for adult patients in clinical settings	2015
PneumaCare Limited (Cambridge, UK) [https://www.accessdata.fda.gov/cdrh_docs/pdf15/K151940.pdf accessed on 22 November 2024]	Thora-3Di, Model T-01 [Structured light plethysmography imaging]	±0.75 BPM (8–25 BPM)	Spot measurements Intended for adult patients in clinical settings	2016
Skanray Technologies Pvt Ltd. (Mysuru, Karnataka, India) [https://www.accessdata.fda.gov/cdrh_docs/pdf17/K172147.pdf accessed on 22 November 2024]	Star 65 [ECG, Capnography]	±1 BPM (0–30 BPM) ±2 BPM (30–60 BPM) ±4 BPM (60–150 BPM)	Continuous monitoring Intended for adults, pediatrics, neonates—Clinical environments	2018
